# 4-(2-Iodo­benzene­sulfonamido)benzoic acid monohydrate

**DOI:** 10.1107/S1600536808043754

**Published:** 2009-01-08

**Authors:** Muhammad Nadeem Arshad, M. Nawaz Tahir, Islam Ullah Khan, Waseeq Ahmad Siddiqui, Muhammad Shafiq

**Affiliations:** a Department of Chemistry, Government College University, Lahore, Pakistan; b Department of Physics, University of Sargodha, Sagrodha, Pakistan; c Department of Chemistry, University of Sargodha, Sagrodha, Pakistan

## Abstract

In the mol­ecule of the title compound, C_13_H_10_INO_4_S·H_2_O, the coordination around the S atom is distorted tetra­hedral. The aromatic rings are oriented at a dihedral angle of 74.18 (17)°. Intra­molecular C—H⋯O hydrogen bonds result in the formation of non-planar five- and six-membered rings, which adopt envelope and twist conformations, respectively. In the crystal structure, inter­molecular N—H⋯O, O—H⋯O and C—H⋯O hydrogen bonds link the mol­ecules. π–π Contacts between the phenyl rings [centroid–centroid distance = 3.726 (3) Å] may further stabilize the structure. There is also a C—H⋯π inter­action.

## Related literature

For general background, see: Medina *et al.* (1999 ▶). For related structures, see: Arshad *et al.* (2008*a* ▶,*b* ▶); Nan & Xing (2006 ▶); Deng & Mani (2006 ▶).
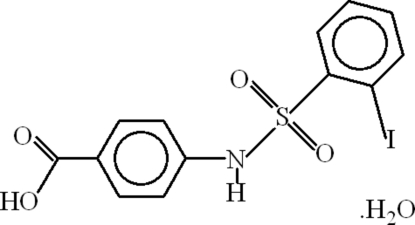


## Experimental

### 

#### Crystal data

C_13_H_10_INO_4_S·H_2_O
*M* *_r_* = 421.20
Monoclinic, 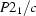

*a* = 13.8049 (9) Å
*b* = 8.2756 (5) Å
*c* = 14.7928 (10) Å
β = 117.472 (3)°
*V* = 1499.42 (17) Å^3^
*Z* = 4
Mo *K*α radiation
μ = 2.30 mm^−1^
*T* = 296 (2) K
0.28 × 0.10 × 0.07 mm

#### Data collection

Bruker Kappa APEXII CCD diffractometer
Absorption correction: multi-scan (*SADABS*; Bruker, 2005 ▶) *T*_min_ = 0.754, *T*_max_ = 0.849
9099 measured reflections
3687 independent reflections
2022 reflections with *I* > 2σ(*I*)
*R*_int_ = 0.041

#### Refinement

*R*[*F* ^2^ > 2σ(*F* ^2^)] = 0.046
*wR*(*F* ^2^) = 0.112
*S* = 1.01
3687 reflections
193 parameters
1 restraint
H atoms treated by a mixture of independent and constrained refinement
Δρ_max_ = 0.55 e Å^−3^
Δρ_min_ = −0.54 e Å^−3^

### 

Data collection: *APEX2* (Bruker, 2007 ▶); cell refinement: *SAINT* (Bruker, 2007 ▶); data reduction: *SAINT*; program(s) used to solve structure: *SHELXS97* (Sheldrick, 2008 ▶); program(s) used to refine structure: *SHELXL97* (Sheldrick, 2008 ▶); molecular graphics: *ORTEP-3 for Windows* (Farrugia, 1997 ▶) and *PLATON* (Spek, 2003 ▶); software used to prepare material for publication: *WinGX* (Farrugia, 1999 ▶) and *PLATON*.

## Supplementary Material

Crystal structure: contains datablocks global, I. DOI: 10.1107/S1600536808043754/hk2605sup1.cif

Structure factors: contains datablocks I. DOI: 10.1107/S1600536808043754/hk2605Isup2.hkl

Additional supplementary materials:  crystallographic information; 3D view; checkCIF report

## Figures and Tables

**Table 1:** Hydrogen-bond geometry (Å, °)

*D*—H⋯*A*	*D*—H	H⋯*A*	*D*⋯*A*	*D*—H⋯*A*
N1—H1⋯O4^i^	0.86	2.03	2.860 (6)	161.00
O3—H3*O*⋯O5^ii^	0.91 (7)	1.73 (7)	2.616 (6)	165 (7)
O5—H5*A*⋯O2^iii^	0.81	2.20	2.924 (6)	149.00
O5—H5*B*⋯O1	0.88	1.98	2.791 (6)	152.00
C6—H6⋯O1	0.93	2.36	2.793 (7)	108.00
C11—H11⋯O2^iv^	0.93	2.52	3.437 (6)	171.00
C12—H12⋯O1	0.93	2.54	3.035 (7)	114.00
C3—H3⋯*Cg*2^v^	0.93	2.90	3.818 (7)	168.00

Symmetry codes: (i) 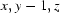; (ii) 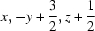; (iii) 
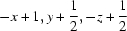
; (iv) 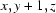; (v) 
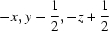
. *Cg*2 is the centroid of the C7–C12 ring.

**Fig. 1.**
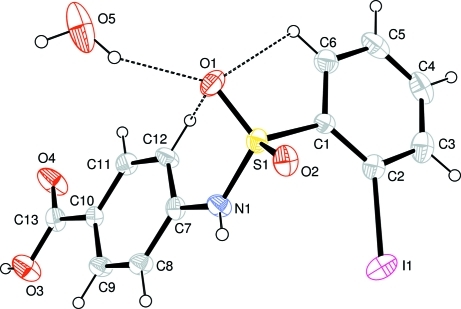
The molecular structure of the title molecule, with the atom-numbering scheme. Displacement ellipsoids are drawn at the 30% probability level. Hydrogen bonds are shown as dashed lines.

**Fig. 2.**
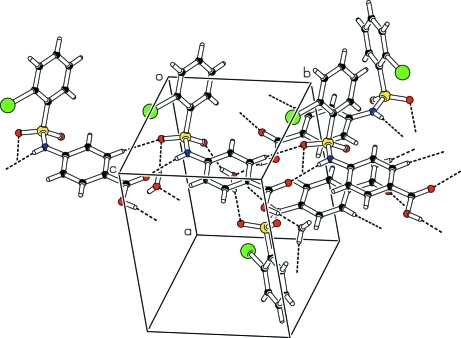
A partial packing diagram of the title compound. Hydrogen bonds are shown as dashed lines.

## supplementary crystallographic information

### Comment

The title compound belongs to the sulfonamide family of the organic compounds. This class of compounds is used as antibecterial agent. The halogenated sulfonamide is used as an inhibitor for the growth of multidrug resistant MCF-7/ADR cancer cells (Medina *et al.*, 1999). In continuation to our researches with sulfonamides (Arshad *et al.*, 2008a,b), the title compound has been prepared, which will be utilized for the syntheses of biologically active heterocyclic molecules with thiazine moiety, and we report herein its crystal structure.

In the title compound, (I), (Fig 1), 2-iodophenyl and *p*-aminobenzoic acid moieties are connected through the SO_2_ group. The structure of (I) differs from 4-(tosylamino)benzoic acid, (II) (Nan & Xing, 2006), mainly due to the attachment of the iodo group at *ortho* position instead of methyl group at the *para*-position. The coordination around the S atom is a distorted tetrahedral. Rings A(C1-C6) and B(C7-C12) are oriented at a dihedral angle of 74.18 (17)°. The intramolecular C-H···O hydrogen bonds (Table 1) result in the formations of nonplanar five- and six-membered rings: C (S1/O1/C1/C6/H6) and D (S1/O1/N1/C7/C12/H12). Ring C adopts envelope conformation with O1 atom displaced by -0.172 (3) Å from the plane of the other rings atoms, while ring D has twisted conformation.

In the crystal structure, intermolecular N-H···O, O-H···O and C-H···O hydrogen bonds (Table 1) link the molecules (Fig. 2), in which they may be effective in the stabilization of the structure. The π-π contact between the phenyl rings, Cg1—Cg1^i^  [symmetry code: (i) -x, -y, -z, where Cg1 is centroid of the ring A (C1-C6)] may further stabilize the structure, with centroid-centroid distance of 3.726 (3) Å. There also exists a C–H···π interaction (Table 1).

### Experimental

The title compound was synthesized according to a literature method (Deng & Mani, 2006). 4-Aminobenzoic acid (0.23 g, 1.67 mmol) was suspended in distilled water (10 ml) in a round bottom flask. The pH of the solution was adjusted to 8-9 using Na_2_CO_3_ (1 M). Then, 2-iodobenzene sulfonyl chloride (0.5 g, 1.66 mmol) was added, and stirred at room temperature. The reaction pH was maintained at 8-9. Completion of reaction was indicated by the dissolvation of the suspended 2-iodobenzene sulfonyl chloride. Then, pH was adjusted to 2-3 using HCl (2 N), the precipitate formed was filtered, washed with distilled water, and then recrystalyzed in methanol.

### Refinement

H3O (for OH) atom was located in difference syntheses and refined [O-H = 0.91 (7) Å, U_iso_(H) = 1.2U_eq_(O)]. The remaining H atoms were positioned geometrically, with O-H = 0.81 and 0.88 Å (for H_2_O), N-H = 0.86 Å (for NH) and C-H = 0.93 Å for aromatic H, respectively, and constrained to ride on their parent atoms with U_iso_(H) = 1.2U_eq_(C,N,O).

### Crystal data

**Table:**

C_13_H_10_INO_4_S·H_2_O	*F*(000) = 824
*M**_r_* = 421.20	*D*_x_ = 1.866 Mg m^−^^3^
Monoclinic, *P*2_1_/*c*	Mo *K*α radiation, λ = 0.71073 Å
Hall symbol: -P 2ybc	Cell parameters from 3638 reflections
*a* = 13.8049 (9) Å	θ = 2.8–28.3°
*b* = 8.2756 (5) Å	µ = 2.30 mm^−^^1^
*c* = 14.7928 (10) Å	*T* = 296 K
β = 117.472 (3)°	Needle, light brown
*V* = 1499.42 (17) Å^3^	0.28 × 0.10 × 0.07 mm
*Z* = 4	

### Data collection

**Table:**

Bruker Kappa APEXII CCD diffractometer	3687 independent reflections
Radiation source: fine-focus sealed tube	2022 reflections with *I* > 2σ(*I*)
graphite	*R*_int_ = 0.041
Detector resolution: 7.40 pixels mm^-1^	θ_max_ = 28.3°, θ_min_ = 2.8°
ω scans	*h* = −15→18
Absorption correction: multi-scan (*SADABS*; Bruker, 2005)	*k* = −11→6
*T*_min_ = 0.754, *T*_max_ = 0.849	*l* = −19→19
9099 measured reflections	

### Refinement

**Table:**

Refinement on *F*^2^	Primary atom site location: structure-invariant direct methods
Least-squares matrix: full	Secondary atom site location: difference Fourier map
*R*[*F*^2^ > 2σ(*F*^2^)] = 0.046	Hydrogen site location: inferred from neighbouring sites
*wR*(*F*^2^) = 0.112	H atoms treated by a mixture of independent and constrained refinement
*S* = 1.01	*w* = 1/[σ^2^(*F*_o_^2^) + (0.0445*P*)^2^] where *P* = (*F*_o_^2^ + 2*F*_c_^2^)/3
3687 reflections	(Δ/σ)_max_ < 0.001
193 parameters	Δρ_max_ = 0.55 e Å^−^^3^
1 restraint	Δρ_min_ = −0.54 e Å^−^^3^

### Special details

**Table:**

Geometry. Bond distances, angles *etc*. have been calculated using the rounded fractional coordinates. All su's are estimated from the variances of the (full) variance-covariance matrix. The cell e.s.d.'s are taken into account in the estimation of distances, angles and torsion angles
Refinement. Refinement of *F*^2^ against ALL reflections. The weighted *R*-factor *wR* and goodness of fit *S* are based on *F*^2^, conventional *R*-factors *R* are based on *F*, with *F* set to zero for negative *F*^2^. The threshold expression of *F*^2^ > σ(*F*^2^) is used only for calculating *R*-factors(gt) *etc*. and is not relevant to the choice of reflections for refinement. *R*-factors based on *F*^2^ are statistically about twice as large as those based on *F*, and *R*- factors based on ALL data will be even larger.

### Fractional atomic coordinates and isotropic or equivalent isotropic displacement parameters (Å^2^)

**Table:**

	*x*	*y*	*z*	*U*_iso_*/*U*_eq_	
I1	0.05915 (3)	−0.04749 (5)	0.26394 (3)	0.0542 (2)	
S1	0.25343 (9)	0.13703 (16)	0.19929 (10)	0.0345 (4)	
O1	0.3085 (3)	0.2410 (4)	0.1612 (3)	0.0454 (14)	
O2	0.2710 (3)	−0.0328 (4)	0.2010 (3)	0.0415 (11)	
O3	0.4294 (3)	0.7980 (5)	0.6032 (3)	0.0614 (17)	
O4	0.3555 (4)	0.9284 (5)	0.4580 (3)	0.0710 (17)	
O5	0.4923 (3)	0.4220 (5)	0.1937 (3)	0.086 (2)	
N1	0.2852 (3)	0.1876 (5)	0.3139 (3)	0.0377 (16)	
C1	0.1119 (4)	0.1750 (5)	0.1231 (4)	0.0292 (17)	
C2	0.0316 (4)	0.1050 (6)	0.1405 (4)	0.0363 (19)	
C3	−0.0775 (4)	0.1337 (7)	0.0725 (5)	0.051 (2)	
C4	−0.1052 (5)	0.2319 (8)	−0.0099 (5)	0.056 (2)	
C5	−0.0257 (5)	0.2994 (7)	−0.0279 (4)	0.054 (2)	
C6	0.0834 (4)	0.2707 (6)	0.0389 (4)	0.044 (2)	
C7	0.3051 (3)	0.3426 (6)	0.3580 (4)	0.0331 (18)	
C8	0.3537 (4)	0.3529 (6)	0.4634 (4)	0.0392 (19)	
C9	0.3778 (4)	0.5001 (6)	0.5106 (4)	0.0378 (17)	
C10	0.3543 (3)	0.6425 (6)	0.4554 (4)	0.0327 (16)	
C11	0.3061 (4)	0.6315 (6)	0.3498 (4)	0.0391 (19)	
C12	0.2810 (4)	0.4842 (6)	0.3012 (4)	0.0397 (17)	
C13	0.3791 (4)	0.8042 (7)	0.5038 (5)	0.0417 (19)	
H1	0.29097	0.10916	0.35421	0.0453*	
H3	−0.13213	0.08538	0.08334	0.0614*	
H3O	0.440 (5)	0.896 (8)	0.634 (5)	0.0734*	
H4	−0.17827	0.25271	−0.05378	0.0676*	
H5	−0.04448	0.36446	−0.08481	0.0649*	
H6	0.13757	0.31670	0.02652	0.0534*	
H8	0.36997	0.25903	0.50216	0.0469*	
H9	0.41064	0.50465	0.58138	0.0457*	
H11	0.29045	0.72558	0.31126	0.0474*	
H12	0.24791	0.47936	0.23047	0.0474*	
H5A	0.55225	0.43714	0.24163	0.1030*	
H5B	0.44825	0.35754	0.20513	0.1030*	

### Atomic displacement parameters (Å^2^)

**Table:**

	*U*^11^	*U*^22^	*U*^33^	*U*^12^	*U*^13^	*U*^23^
I1	0.0626 (3)	0.0469 (3)	0.0680 (3)	0.0006 (2)	0.0429 (2)	0.0116 (2)
S1	0.0343 (6)	0.0313 (8)	0.0384 (9)	−0.0025 (6)	0.0171 (6)	−0.0039 (6)
O1	0.049 (2)	0.043 (2)	0.056 (3)	−0.0132 (17)	0.0343 (19)	−0.0061 (19)
O2	0.0403 (19)	0.029 (2)	0.050 (2)	0.0031 (16)	0.0165 (17)	−0.0062 (18)
O3	0.086 (3)	0.032 (3)	0.043 (3)	0.003 (2)	0.010 (2)	−0.007 (2)
O4	0.105 (3)	0.027 (3)	0.045 (3)	0.005 (2)	0.004 (2)	0.005 (2)
O5	0.046 (2)	0.076 (4)	0.100 (4)	−0.015 (2)	0.003 (2)	0.054 (3)
N1	0.053 (3)	0.023 (2)	0.029 (3)	−0.0021 (19)	0.012 (2)	0.003 (2)
C1	0.035 (3)	0.024 (3)	0.028 (3)	0.002 (2)	0.014 (2)	−0.001 (2)
C2	0.039 (3)	0.028 (3)	0.044 (4)	0.002 (2)	0.021 (3)	−0.007 (2)
C3	0.043 (3)	0.042 (4)	0.070 (5)	−0.003 (3)	0.027 (3)	−0.016 (3)
C4	0.046 (3)	0.051 (4)	0.049 (4)	0.008 (3)	0.002 (3)	−0.011 (3)
C5	0.073 (4)	0.041 (4)	0.032 (4)	0.009 (3)	0.010 (3)	−0.001 (3)
C6	0.054 (3)	0.037 (4)	0.040 (4)	−0.005 (3)	0.020 (3)	−0.003 (3)
C7	0.030 (2)	0.028 (3)	0.038 (4)	0.000 (2)	0.013 (2)	−0.004 (3)
C8	0.052 (3)	0.023 (3)	0.041 (4)	0.001 (2)	0.020 (3)	0.006 (3)
C9	0.050 (3)	0.028 (3)	0.033 (3)	0.000 (2)	0.017 (3)	0.001 (2)
C10	0.028 (2)	0.032 (3)	0.035 (3)	−0.002 (2)	0.012 (2)	−0.002 (3)
C11	0.044 (3)	0.023 (3)	0.046 (4)	0.000 (2)	0.017 (3)	0.007 (3)
C12	0.050 (3)	0.033 (3)	0.027 (3)	0.001 (2)	0.010 (3)	0.003 (2)
C13	0.036 (3)	0.030 (3)	0.048 (4)	0.004 (2)	0.010 (3)	0.002 (3)

### Geometric parameters (Å, °)

**Table:**

I1—C2	2.105 (5)	C5—C6	1.389 (9)
S1—O1	1.425 (4)	C7—C8	1.387 (7)
S1—O2	1.425 (4)	C7—C12	1.390 (7)
S1—N1	1.599 (4)	C8—C9	1.367 (7)
S1—C1	1.776 (6)	C9—C10	1.384 (7)
O3—C13	1.306 (8)	C10—C13	1.481 (8)
O4—C13	1.191 (7)	C10—C11	1.390 (7)
O3—H3O	0.91 (7)	C11—C12	1.376 (7)
O5—H5A	0.8100	C3—H3	0.9300
O5—H5B	0.8800	C4—H4	0.9300
N1—C7	1.408 (6)	C5—H5	0.9300
N1—H1	0.8600	C6—H6	0.9300
C1—C2	1.376 (8)	C8—H8	0.9300
C1—C6	1.372 (7)	C9—H9	0.9300
C2—C3	1.392 (9)	C11—H11	0.9300
C3—C4	1.365 (9)	C12—H12	0.9300
C4—C5	1.363 (10)		
			
I1···O2	3.456 (5)	C4···O2^iv^	3.158 (8)
I1···N1	3.456 (4)	C4···I1^xi^	3.853 (7)
I1···C4^i^	3.853 (7)	C5···C5^xii^	3.416 (8)
I1···C2^i^	3.671 (5)	C8···O1^xiii^	3.353 (7)
I1···C3^i^	3.509 (6)	C9···C9^x^	3.530 (9)
I1···H1	3.1200	C12···O1	3.035 (7)
S1···H12	2.8800	C13···O5^v^	3.379 (7)
S1···H5A^ii^	2.9200	C9···H3^xi^	3.1000
O1···O5	2.791 (6)	C10···H3^xi^	2.8900
O1···C12	3.035 (7)	C11···H3^xi^	3.0100
O1···C8^iii^	3.353 (7)	H1···I1	3.1200
O2···C4^iv^	3.158 (8)	H1···H8	2.3100
O2···O5^ii^	2.924 (6)	H1···O4^ix^	2.0300
O2···I1	3.456 (5)	H3···C10^i^	2.8900
O3···O5^v^	2.616 (6)	H3···C9^i^	3.1000
O4···N1^vi^	2.860 (6)	H3···C11^i^	3.0100
O5···O3^vii^	2.616 (6)	H3O···O5^v^	1.73 (7)
O5···C13^vii^	3.379 (7)	H3O···H5A^v^	2.1400
O5···O2^viii^	2.924 (6)	H3O···H5B^v^	2.2700
O5···O1	2.791 (6)	H4···O2^iv^	2.6700
O1···H12	2.5400	H5A···O2^viii^	2.2000
O1···H5B	1.9800	H5A···H9^x^	2.4700
O1···H6	2.3600	H5A···S1^viii^	2.9200
O1···H8^iii^	2.8500	H5A···H3O^vii^	2.1400
O2···H4^iv^	2.6700	H5B···O1	1.9800
O2···H11^ix^	2.5200	H5B···O3^x^	2.8500
O2···H5A^ii^	2.2000	H5B···H3O^vii^	2.2700
O3···H9	2.4500	H6···O1	2.3600
O3···H5B^x^	2.8500	H8···O4^ix^	2.8000
O4···H1^vi^	2.0300	H8···H1	2.3100
O4···H8^vi^	2.8000	H8···O1^xiii^	2.8500
O4···H11	2.5600	H9···O3	2.4500
O5···H3O^vii^	1.73 (7)	H9···H5A^x^	2.4700
N1···O4^ix^	2.860 (6)	H11···O2^vi^	2.5200
N1···I1	3.456 (4)	H11···O4	2.5600
C2···I1^xi^	3.671 (5)	H12···O1	2.5400
C3···I1^xi^	3.509 (6)	H12···S1	2.8800
			
O1—S1—O2	119.0 (3)	C8—C9—C10	121.5 (5)
O1—S1—N1	109.0 (2)	C9—C10—C11	117.9 (5)
O1—S1—C1	105.9 (2)	C11—C10—C13	119.1 (5)
O2—S1—N1	106.2 (2)	C9—C10—C13	123.0 (5)
O2—S1—C1	108.3 (2)	C10—C11—C12	121.3 (5)
N1—S1—C1	108.1 (3)	C7—C12—C11	119.9 (5)
C13—O3—H3O	114 (4)	O3—C13—C10	113.2 (5)
H5A—O5—H5B	116.00	O4—C13—C10	124.3 (6)
S1—N1—C7	129.0 (4)	O3—C13—O4	122.6 (6)
C7—N1—H1	116.00	C2—C3—H3	120.00
S1—N1—H1	115.00	C4—C3—H3	120.00
S1—C1—C2	123.5 (4)	C5—C4—H4	120.00
S1—C1—C6	116.7 (5)	C3—C4—H4	120.00
C2—C1—C6	119.6 (5)	C4—C5—H5	120.00
I1—C2—C1	125.2 (4)	C6—C5—H5	120.00
C1—C2—C3	119.3 (5)	C5—C6—H6	120.00
I1—C2—C3	115.6 (4)	C1—C6—H6	120.00
C2—C3—C4	120.7 (6)	C7—C8—H8	120.00
C3—C4—C5	120.0 (6)	C9—C8—H8	120.00
C4—C5—C6	119.9 (5)	C10—C9—H9	119.00
C1—C6—C5	120.5 (6)	C8—C9—H9	119.00
C8—C7—C12	119.0 (5)	C10—C11—H11	119.00
N1—C7—C8	117.8 (4)	C12—C11—H11	119.00
N1—C7—C12	123.2 (5)	C7—C12—H12	120.00
C7—C8—C9	120.4 (5)	C11—C12—H12	120.00
			
O1—S1—N1—C7	35.5 (5)	C1—C2—C3—C4	−0.9 (9)
O2—S1—N1—C7	164.9 (5)	C2—C3—C4—C5	1.9 (10)
C1—S1—N1—C7	−79.1 (5)	C3—C4—C5—C6	−1.3 (9)
O1—S1—C1—C2	−175.7 (4)	C4—C5—C6—C1	−0.1 (8)
O1—S1—C1—C6	9.1 (4)	N1—C7—C8—C9	178.2 (5)
O2—S1—C1—C2	55.6 (5)	C12—C7—C8—C9	−0.2 (9)
O2—S1—C1—C6	−119.7 (4)	N1—C7—C12—C11	−177.8 (5)
N1—S1—C1—C2	−59.0 (5)	C8—C7—C12—C11	0.4 (9)
N1—S1—C1—C6	125.7 (4)	C7—C8—C9—C10	0.3 (9)
S1—N1—C7—C8	−166.5 (4)	C8—C9—C10—C11	−0.6 (9)
S1—N1—C7—C12	11.8 (8)	C8—C9—C10—C13	180.0 (6)
S1—C1—C2—I1	4.0 (6)	C9—C10—C11—C12	0.9 (9)
S1—C1—C2—C3	−175.7 (4)	C13—C10—C11—C12	−179.7 (6)
C6—C1—C2—I1	179.1 (4)	C9—C10—C13—O3	2.8 (8)
C6—C1—C2—C3	−0.5 (8)	C9—C10—C13—O4	−176.6 (6)
S1—C1—C6—C5	176.5 (4)	C11—C10—C13—O3	−176.6 (5)
C2—C1—C6—C5	1.0 (8)	C11—C10—C13—O4	4.0 (9)
I1—C2—C3—C4	179.4 (5)	C10—C11—C12—C7	−0.8 (9)

Symmetry codes: (i) −*x*, *y*−1/2, −*z*+1/2; (ii) −*x*+1, *y*−1/2, −*z*+1/2; (iii) *x*, −*y*+1/2, *z*−1/2; (iv) −*x*, −*y*, −*z*; (v) *x*, −*y*+3/2, *z*+1/2; (vi) *x*, *y*+1, *z*; (vii) *x*, −*y*+3/2, *z*−1/2; (viii) −*x*+1, *y*+1/2, −*z*+1/2; (ix) *x*, *y*−1, *z*; (x) −*x*+1, −*y*+1, −*z*+1; (xi) −*x*, *y*+1/2, −*z*+1/2; (xii) −*x*, −*y*+1, −*z*; (xiii) *x*, −*y*+1/2, *z*+1/2.

### Hydrogen-bond geometry (Å, °)

**Table:**

*D*—H···*A*	*D*—H	H···*A*	*D*···*A*	*D*—H···*A*
N1—H1···O4^ix^	0.86	2.03	2.860 (6)	161.00
O3—H3O···O5^v^	0.91 (7)	1.73 (7)	2.616 (6)	165 (7)
O5—H5A···O2^viii^	0.81	2.20	2.924 (6)	149.00
O5—H5B···O1	0.88	1.98	2.791 (6)	152.00
C6—H6···O1	0.93	2.36	2.793 (7)	108.00
C11—H11···O2^vi^	0.93	2.52	3.437 (6)	171.00
C12—H12···O1	0.93	2.54	3.035 (7)	114.00
C3—H3···Cg2^i^	0.93	2.90	3.818 (7)	168.00

Symmetry codes: (ix) *x*, *y*−1, *z*; (v) *x*, −*y*+3/2, *z*+1/2; (viii) −*x*+1, *y*+1/2, −*z*+1/2; (vi) *x*, *y*+1, *z*; (i) −*x*, *y*−1/2, −*z*+1/2.
